# What Is the Ability Emotional Intelligence Test (MSCEIT) Good for? An Evaluation Using Item Response Theory

**DOI:** 10.1371/journal.pone.0098827

**Published:** 2014-06-05

**Authors:** Marina Fiori, Jean-Philippe Antonietti, Moira Mikolajczak, Olivier Luminet, Michel Hansenne, Jérôme Rossier

**Affiliations:** 1 Department of Organizational Behavior, Faculty of Business and Economics, University of Lausanne, Lausanne, Switzerland; 2 Institute of Psychology, Faculty of Social and Political Sciences, University of Lausanne, Lausanne, Switzerland; 3 Department of Psychology, Catholic University of Louvain, Louvain-la-Neuve, Belgium; 4 Department of Psychology, University of Liège, Liège, Belgium; University of São Paulo, Brazil

## Abstract

The ability approach has been indicated as promising for advancing research in emotional intelligence (EI). However, there is scarcity of tests measuring EI as a form of intelligence. The Mayer Salovey Caruso Emotional Intelligence Test, or MSCEIT, is among the few available and the most widespread measure of EI as an ability. This implies that conclusions about the value of EI as a meaningful construct and about its utility in predicting various outcomes mainly rely on the properties of this test. We tested whether individuals who have the highest probability of choosing the most correct response on any item of the test are also those who have the strongest EI ability. Results showed that this is *not* the case for most items: The answer indicated by experts as the most correct in several cases was not associated with the highest ability; furthermore, items appeared too easy to challenge individuals high in EI. Overall results suggest that the MSCEIT is best suited to discriminate persons at the low end of the trait. Results are discussed in light of applied and theoretical considerations.

## Introduction

Nearly two decades ago emotional intelligence entered the scene of psychological inquiry and since then it has increasingly gained a place in the scientific community. Emotional Intelligence (EI) can be defined as the capacity to recognize emotions in oneself and others, understand how they originate, develop, and change during emotional experience, and use this understanding to enhance thinking and behavior. Two conceptually different approaches have been developed to study EI: the trait and the ability approach [1]. The first conceives EI as a dispositional tendency, such as a personality trait, and measures the construct with self-report questionnaires; the second conceptualizes EI as an ability based on the processing of emotion information and assesses it with performance tests. This article concerns the latter approach of EI; more specifically, it presents a contribution regarding the most well-known and employed test to measure EI as an ability: The Mayer Salovey Caruso Emotional Intelligence Test or MSCEIT [2].

The ability EI approach was introduced in its original formulation by Salovey and Mayer [3]. It received encouraging feedback throughout the years [4] and was considered worth pursuing even by skeptics of the EI concept (e.g. [5]). Although the ability approach shows promise, several issues still remain unsettled. Research has yet to demonstrate the extent to which ability EI is distinct from other existing constructs - such as personality and general intelligence - and how it accounts for emotionally intelligent performance. For instance, the personality trait of agreeableness predicted a substantial amount of variance in EI scales (e.g. [6], [7]).

Furthermore, several studies failed to find an association between ability EI scales and emotion information processing, showing that current measures of EI may be tapping into just one aspect of intelligence, namely crystallized intelligence [8], [9], [10].

A fundamental issue that has not received adequate attention from EI scholars refers to better understanding what aspect of EI current ability tests measure and with what level of accuracy. In fact, there is scarcity of tests measuring EI as a form of intelligence. The Mayer Salovey Caruso Emotional Intelligence Test (MSCEIT) is among the very few available and the most well-known and accepted measure of EI as an ability. Thus, conclusions about EI as a meaningful construct and of its utility in predicting various outcomes mostly rely on the properties of this test. Hence, discerning whether a test of EI can be trusted, and to what extent, is of primary importance for advancing research in this domain.

We aimed to provide a contribution on the quality of ability EI tests by analyzing the MSCEIT from a perspective that is relatively new to the domain of EI: the Item Response Theory (IRT). We opted for such an approach because it allows for investigating the properties of this test at the item level and because it provides a different evaluation of the test than classical test theory (CTT). For instance, CTT assumes that the measurement precision of a test remains constant along the underlying latent trait. With IRT we aimed to test whether this assumption holds true for the MSCEIT along the different trait levels so as to understand whether this test is a good tool for discriminating individuals along the ability EI trait, especially those placed at the higher end of the distribution.

## Measuring EI as an Ability

Ability EI tests differ greatly from self-report measures of EI because they are based on the analysis of how individuals perform at their best in certain conditions (maximal performance) instead of assessing how individuals perform on a daily basis (typical performance). Furthermore, in ability EI tests, correctness of responses is not evaluated by the subject him/herself, as it is the case for personality questionnaires, but it is determined on the basis of an external criterion of correctness. The issue of how to establish a correct answer in the domain of emotional intelligence has been (and still remains) the most difficult conundrum to address. Among the most problematic aspect there is how to determine the *one best way* of using/feeling emotions across individuals, given that individuals may differ with respect to how they feel and manage emotions effectively. Furthermore, correctness of emotional reactions may depend on the frame of reference for judging a response as correct. For example, suppressing anger when receiving a negative feedback from the supervisor may be an effective way to manage emotions if the goal of the person is to preserve a good relationship with the boss. However, it may not be considered as an effective reaction if the criterion is to maintain self-esteem and reduce frustration.

How did the authors of the EI test address the issue of scoring the test with respect to an allegedly correct response? Appealing to the idea that emotions are biologically determined (and therefore also shared by all human beings) Mayer, Caruso, and Salovey [11] proposed to score a correct answer according to the response chosen by the majority of people. For example, if a person chooses an answer that was also chosen by 75% of the respondents, then that person obtains a score of.75. The problem residing in the logic of this scoring system appears particularly evident when answers are easy to endorse. In fact, in the case of an easy to endorse answer, most people will get the highest score for a question that is, in fact, easy (i.e. most people identify the correct answer). Furthermore, as noted by MacCann and colleagues [12], if the test is internally consistent and reliable, then the majority of people who score high on an item tend to score high also on other items, especially when items are on average rather easy. The result is that the distribution of the test scores tends to be skewed toward the high end of the distribution, with average and above average EI individuals constituting the peak of the distribution.

Notably, the authors of the MSCEIT also proposed a second scoring system: the expert-based scoring. In this case the correct answer is identified according to the responses provided by the majority of a pool of emotion experts. Mayer, Caruso, and Salovey [11] encouraged using the consensus-based instead of the expert-based scoring because considered more reliable. In any case, the expert scoring does not seem to provide an alternative to the skewedness issue in that, as indicated by the test authors, correlation between the two scoring systems are as high as.99, showing that the experts’ opinion does not diverge much from that of the majority of people, as also recognized by the authors themselves [13].

The concerns previously expressed on the scoring systems of the MSCEIT motivated the current analysis. We decided to investigate the MSCEIT at the item level to check how appropriate they are to measure EI and to discriminate individuals along the EI trait. In particular, we employed latent trait models and analyzed individuals’ responses to items in relation to the properties of the items as well as the position of the individual along the latent trait. Importantly, with Item Response Theory we were able to understand whether the precision of the MSCEIT changed along the latent trait, challenging the assumption of Classical Test Theory that this precision remains constant.

## The MSCEIT

The Mayer Salovey Caruso Emotional Intelligence Test was the first test introduced to measure EI as an ability. Since its very first appearance in 2000 (the test at that time was called Multifactor Emotional Intelligence Scale or MEIS) the MSCEIT has undergone several revisions. The current structure of the test reflects the four-branch model of EI of Mayer and Salovey [14] according to which EI is arranged in a hierarchical structure with one global underlying factor, EI, and 4 abilities or branches: Perceiving Emotions, Using Emotions to Facilitate Thinking, Understanding Emotions, and Managing Emotions. In addition to the theoretical model, the test also includes an intermediate level in which the first two branches are merged into an Experiential Area score and the second two branches into a Strategic Area score. Notably, the authors have recommended using the global score of the MSCEIT in view of the fact that the test measures “one unique source of variation” ([15] p.508).

In addition, each branch is measured through two subscales: Perceiving Emotions includes identifying emotions conveyed through facial expressions and abstract pictures; Using Emotions includes items referring to evaluating how certain moods may facilitate thinking processes and the comparison of emotions to sensations, such as color, light, and temperature; Understanding Emotions includes two subscales that refer to blending emotions and acknowledging how emotions may change and develop; Managing Emotions includes two subscales that refer to rating which emotional strategy would be most appropriate to manage emotions for oneself and with respect to using emotions in interpersonal relationships. All the 141 items included in the test are answered through a Likert-type scale from 1 (not at all present/not at all effective) to 5 (very much present/effective). The MSCEIT was presented as a valid measure of EI [16], [17], [18] although some doubts about its validity were raised in the past (e.g. [19]) and have become more compelling in recent years (e.g. [6], [9], [20], [21]).

## An IRT Approach to the MSCEIT

Item Response Theory denotes a set of mathematical models in which the probability of endorsing a certain response to an item is modeled as a function of the characteristics of the item as well as the respondent’s position along the latent trait. Whereas Classical Test Theory (CTT) has the whole test as the unit of analysis, IRT models provide a way of measuring the quality of a test by analyzing single items, looking into how appropriate they are for discriminating respondents, and testing how well such items measure respondents’ underlying ability/trait. Another important advantage of IRT over CTT is that it may measure the precision of a scale without assuming that it remains constant along the underlying latent trait.

IRT applications to the domain of EI are rather scarce. Cooper and Petrides [22] employed IRT to assess the psychometric properties of the short form of the trait EI test (TEIQue-SF; [23]). The questionnaire showed good precision in discriminating individuals along the trait and high information values for most items. Regarding ability EI, Maul [24] conducted an item analysis of the MSCEIT to investigate the hypothesized structure of the test. He found no strong evidence for preferring a unidimensional model over a four-dimensional model of EI when controlling for facet-related variance. Importantly, no research to date has employed IRT to understand whether the MSCEIT can be trusted as an ability test that discriminates among individuals along the EI trait.

To conduct the analysis we chose unidimensional models of the Rasch family, which assume that items have an equal relationship with the underlying trait and estimate for all items a common discrimination parameter. The simplest Rasch model is the one-parameter logistic (1PL) model in which the probability P of endorsing a correct answer is calculated as a function of the latent trait theta (θ) and the characteristic of the item i, such that for each person j: Pij (θj, bi). More specifically, in this model the b parameter denotes the item difficulty, which corresponds to the point on the latent trait in which the person has 50% chance of responding correctly to the question. We preferred Rasch models because of their parsimony: they are relatively simple models and appeared to fit the data rather well. Furthermore, we used a partial credit Rasch model because the MSCEIT has multiple answers that are scored along a continuum from the most to the least correct answer.

**Figure 1:**
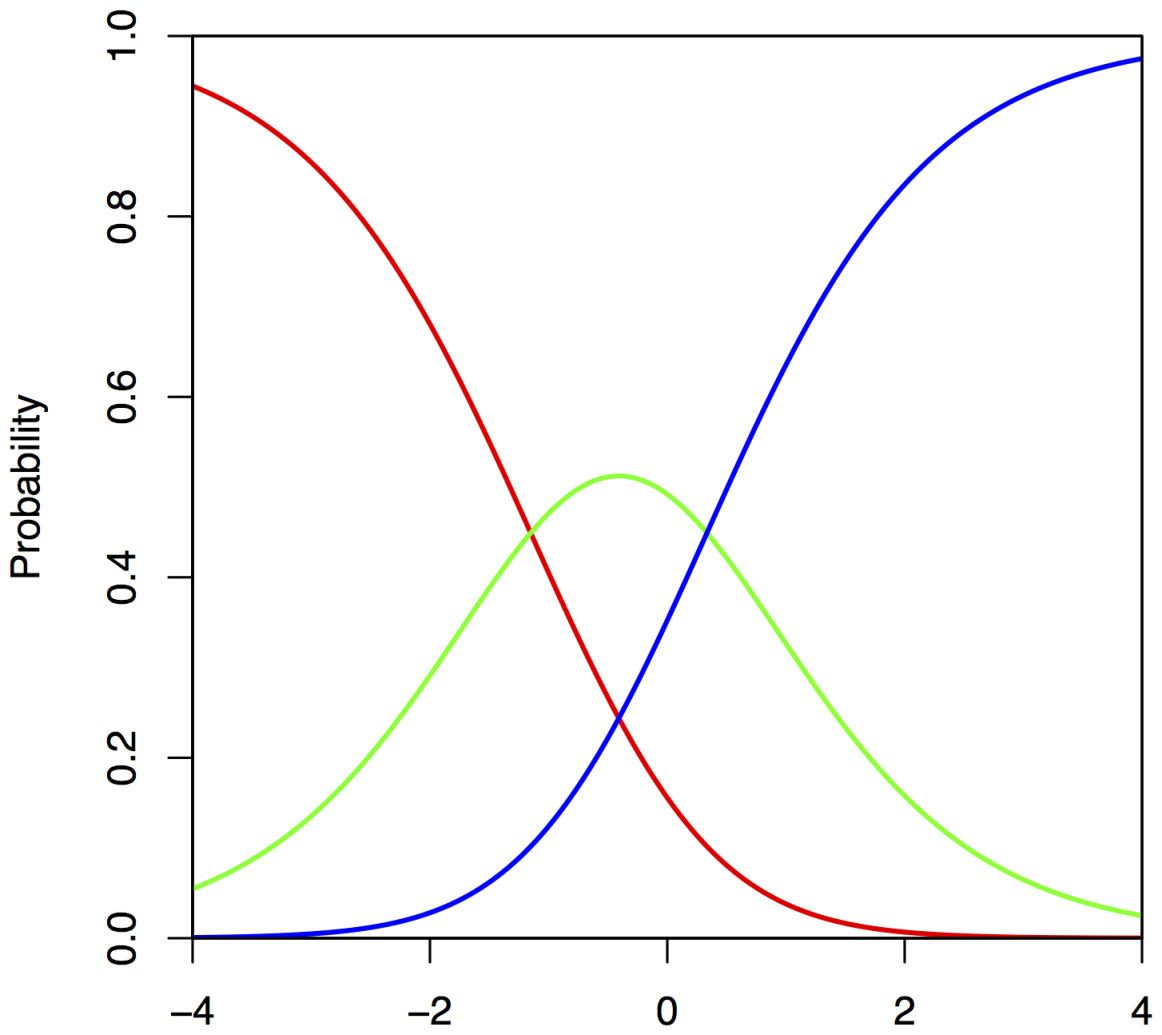
Example of Category Response Curve for the three scoring options employed in the current analysis.

Figure 1 depicts item responses through three probability curves: the red curve corresponds to the probability of choosing the wrong response, coded as 0; the green curve corresponds to the probability of choosing a partially correct answer, coded as 1; the blue curve corresponds to the probability of choosing the most correct answer, coded as 2. When the ability of the subject is low, then the most likely answer is a wrong answer (on the left side of the graph, the red curve prevails). When the ability of the subject is average, then it is more likely that the person will chose a partially correct answer (the central part of the graph is mostly taken by the green line). When the ability of the subject is high, then it is very likely that the person will provide the most correct answer (the right part of the graph is mostly occupied by the blue line).

To explore whether items are equally good at distinguishing individuals on the latent trait, we calculated the item information function (IIF), which indicates the amount of information yielded by each item. This feature of IRT is particularly helpful to determine the precision of measurement of individuals at different levels of the underlying trait. For most models, the amount of information provided by each item is maximized when the difficulty of the item approaches the latent trait of the person. Because item information calculated according to the Rasch model tends to be the same for all items given the constraint of equal discrimination, to calculate IIF we decided to employ a more complex model. We therefore conducted IFF analyses with a 2 PL model in which we allowed item discrimination to vary from item to item.

In addition to estimating the item parameters, we also estimated respondents’ ability using a Bayesian procedure. For each subject we estimated the theta distribution apriori. Then we summarized this distribution a posteriori through its mean. Bock and Mislevy [25] proposed a way to calculate the aposteriori expectation of theta based on a apriori distribution obtained from the data. They called the estimation resulting from this procedure estimation EAP (expected a posteriori). For most of the analyses we employed the software ConQuest [26] that estimates the latent trait using marginal maximum likelihood estimation (MMLE) in which item parameters are treated as fixed effects and ability parameters as random effects. Model fit was tested with residual-based methods developed by Wu [27]. To calculate additional functions, such as the Item Information Function, we also employed the package ltm [28].

## Method

### Participants and Procedure

The sample consisted of 729 participants from the French speaking part of Belgium; 408 were women and 321 men. The mean age was 33.29 years (SD = 12.55). The sample included 152 undergraduate students who were enrolled in psychological courses. The other participants consisted of student's acquaintances. They were recruited by asking students to have the MSCEIT completed by friends and relatives in the framework of a course assignment.

### Ethics Statement

Participation in the study was voluntary and participants could quit the study at any time they wished. The IRB approval was not required at the time the study was conducted.

### Measure

We employed the French version of the Mayer, Salovey, and Caruso Emotional Intelligence Test (MSCEIT) version 2.0, which was filled out online. The test assesses EI with 141 items that are organized in 4 characteristics or ‘Branches’: Perceiving Emotions, which is measured through 2 subscales (sections A and E) referring to identifying emotions conveyed through facial expressions and pictures; Using Emotions, which includes 2 subscales (sections B and F) that refer to how emotions may be employed in different situations and how they may be associated with sensations, such as hot/cold; Understanding Emotions, which includes 2 subscales (sections C and G) referring to understanding the results of combinations of emotions and knowing how emotions may change and develop; Managing Emotions, which includes 2 subscales (section D and H) referred to rating which emotional strategy would be most effective for regulating the self and other people’s emotions. For each item participants indicated the level of effectiveness of a list of options, ranging from 1 = very ineffective to 5 = very effective, or the presence of a certain emotion, ranging from 1 = not at all present to 5 = present to a great extent. Correct answers were scored according to agreement with expert opinion. The test internal consistency reliability (split-half), as indicated in the manual, is r = .93 [29].

## Results

### Descriptive Statistics

We conducted a first analysis on the distribution of responses for each item. The Shapiro-Wilk test showed that all the 141 items of the MSCEIT have a significantly skewed distribution. Interestingly, for the two sections of Branch 1 Perceiving Emotions, the most common answer was 1, which corresponds to absence of any emotion (the Likert scale goes from 1 = not at all present to 5 = present to a great extent). More specifically, response 1 was the most common answer for 15 out of 20 items of section A, and for 28 out of 30 items for section E. This implies that for this branch of the test (Perceiving Emotions) individuals obtain the highest score for, ironically, detecting the presence of ‘no emotions’.

Taking the raw score of each item, we calculated the correlation between the scoring of experts (expert scoring) and that based on the majority of respondents (consensus scoring). For 12 out of 141 items the correlation was either negative or close to 0, showing that experts and common people chose different correct answers on those few items. For 97/141 items the correlation was higher than .90. If taken at the level of the sub-dimensions and branches, correlations between the two scoring systems ranged between .94 and .99. These results show that the two systems provide very similar results and that the issue of the skewedness of responses is common to both scoring systems.

### IRT Analysis

We recoded answers chosen by the majority of experts as ‘2’, answers that were close to the one chosen by the majority of experts as ‘1’, and all other answers as ‘0’. For example, if 4 was the answer chosen by the majority of people, then the score of 4 was recoded as ‘2’, the score of 5 and 3 were recoded as ‘1’, and the score of 1 and 2 were recoded as ‘0’. We employed this coding system to reduce the scoring options from 5 to 3 while maintaining the level of complexity of a partial credit approach.

**Figure 2:**
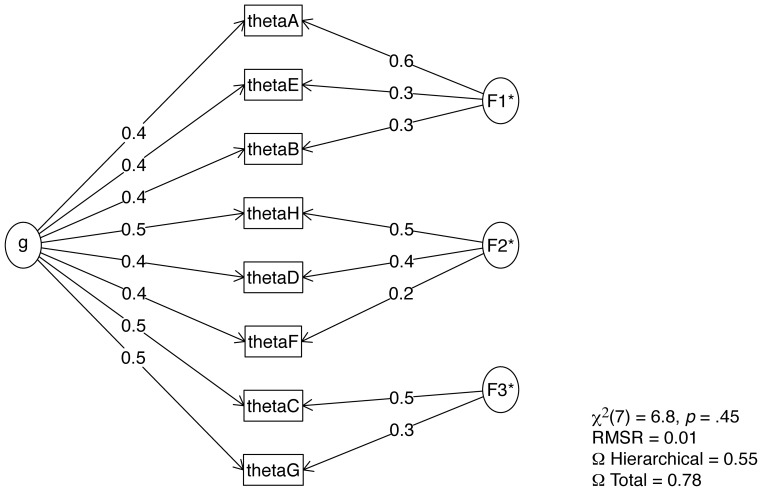
Factorial structure of the MSCEIT’s sub dimensions and results of McDonald’s omega.

We calculated scores on the 8 MSCEIT’s sub-dimensions based on the recoded answers (theta scores); then we calculated McDonald’s omega [30] to estimate the general factor saturation of the test and to check for the unidimensionality requirement of IRT. McDonald’s omega describes the ratio of the variance due to a common factor to the total variance. Results shows that a general factor explains 55% of the variance, and that introducing 3 intermediate factors to the model adds up 23% of variance. Although the latter model is better, the former still appears to fit the data rather well (see Figure 2).

Parameter-level fit statistics, in particular the infit and outfit mean squares, were investigated. These indexes provide information regarding the extent to which the data fit the model. Values are expected to be close to 1; values lower than 1 indicate model redundancy, whereas values higher than 1 indicate that the model is under predicted. Fit indexes did not show any particular misfit for most items, with the exception of 12 items with unsatisfactory parameters, mainly in the two sections of Branch 1, Perceiving Emotions (see Table 1 for the list of problematic items; results of all items are available upon request). Overall results suggest that our model predicts data quite well.

**Table 1:** Infit and outfit statistics of the items that showed unsatisfactory parameters (e.g., values <3/4 and >4/3).

OUTFIT							
Item	Section	Beta	ES.beta	outfit	T.outfit	infit	T.infit
10	A	−0.66	0.05	0.74	−5.50	0.86	−2.00
11	A	−0.79	0.05	1.34	5.90	1.07	0.90
12	A	−0.15	0.05	1.61	9.80	1.45	8.60
15	A	−0.74	0.05	0.71	−6.00	0.84	−2.20
18	A	−0.69	0.05	0.74	−5.50	0.88	−1.70
79	E	−0.99	0.04	0.66	−7.40	0.85	−1.40
80	E	−0.97	0.04	0.68	−7.00	0.86	−1.30
81	E	0.84	0.04	1.48	7.90	1.38	9.10
88	E	−0.47	0.04	0.72	−5.90	0.83	−2.70
93	E	1.26	0.04	1.76	11.90	1.43	9.50
96	E	1.33	0.04	1.78	12.20	1.42	8.80
141	H	−1.10	0.13	1.67	10.70	1.14	1.10
**INFIT**							
**Item**	**Section**	**Beta**	**ES.beta**	**outfit**	**T.outfit**	**infit**	**T.infit**
12	A	−0.15	0.05	1.61	9.80	1.45	8.60
81	E	0.84	0.04	1.48	7.90	1.38	9.10
93	E	1.26	0.04	1.76	11.90	1.43	9.50
96	E	1.33	0.04	1.78	12.20	1.42	8.80

#### The person-parameter distribution

**Figure 3:**
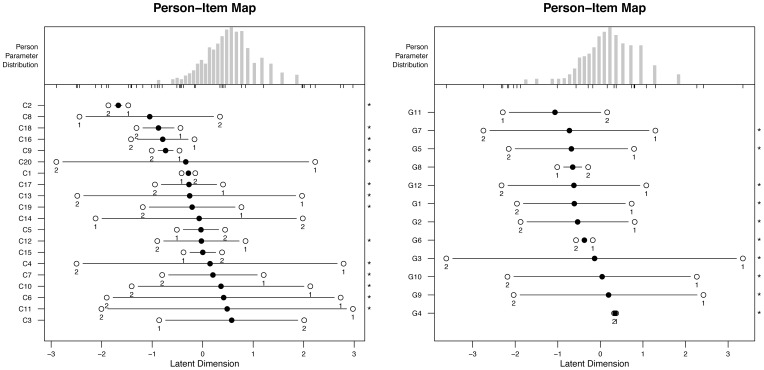
The person’s score on the same metric of the item difficulty for the two sections (C and G) of Branch 3, Perceiving Emotions.

Figure 3 plots the person’s score on the same metric of the item difficulty. As an example, we report scores of the 2 sections of Branch 3 Understanding Emotions (results for all the MSCEIT sections showed the same pattern of Branch 3 and are available upon request). In the figure, the point marked as 1 corresponds to the point in which the probability of responding 0 is equal to the probability of responding 1. The point marked as 2 corresponds to the point in which the probability of responding 1 is equal to the probability of responding 2. When the point marked as 1 is left of point marked as 2, then it means that the most likely answer is the intermediate answer (or answer coded as 1). In contrast, when the point marked as 2 is left of point 1, then it means that the intermediate answer is very unlikely to occur, and that the item functions as if it were dichotomous. Most items show the latter pattern.

#### The person-item map

**Figure 4:**
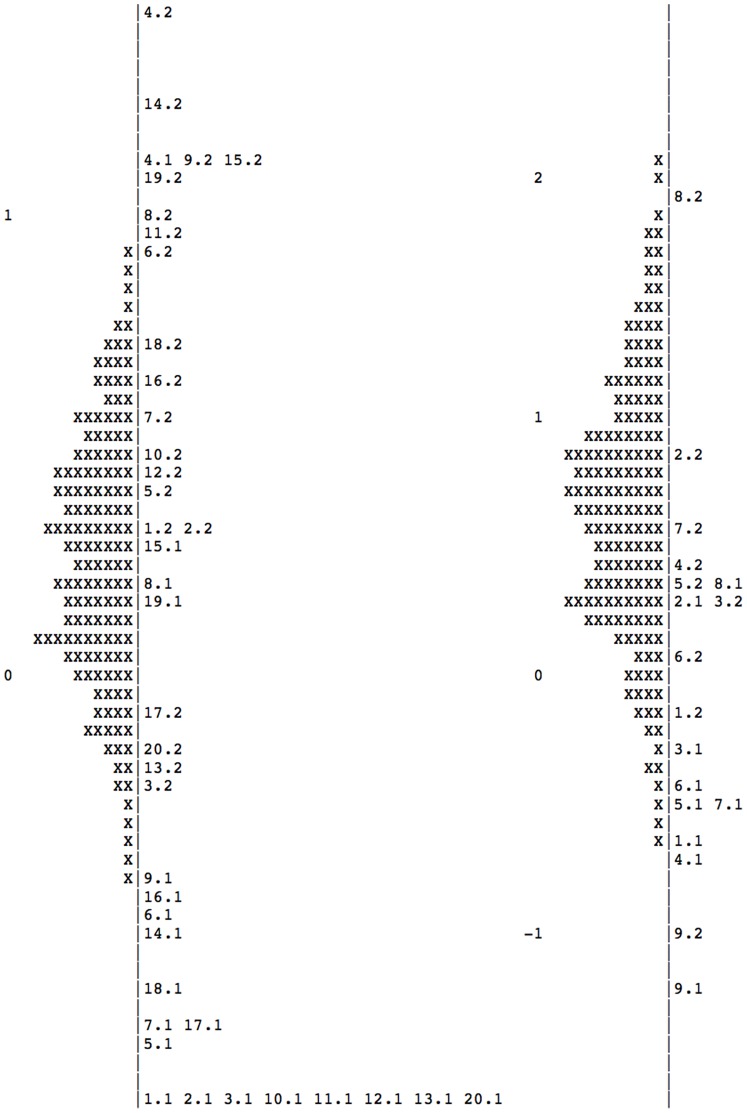
Location of the persons and the items on the same latent trait for the two sections of Branch 4-Managing Emotions: section D (left) and section H (right). Each ‘X’ represents 4.7 cases.

**Figure 5:**
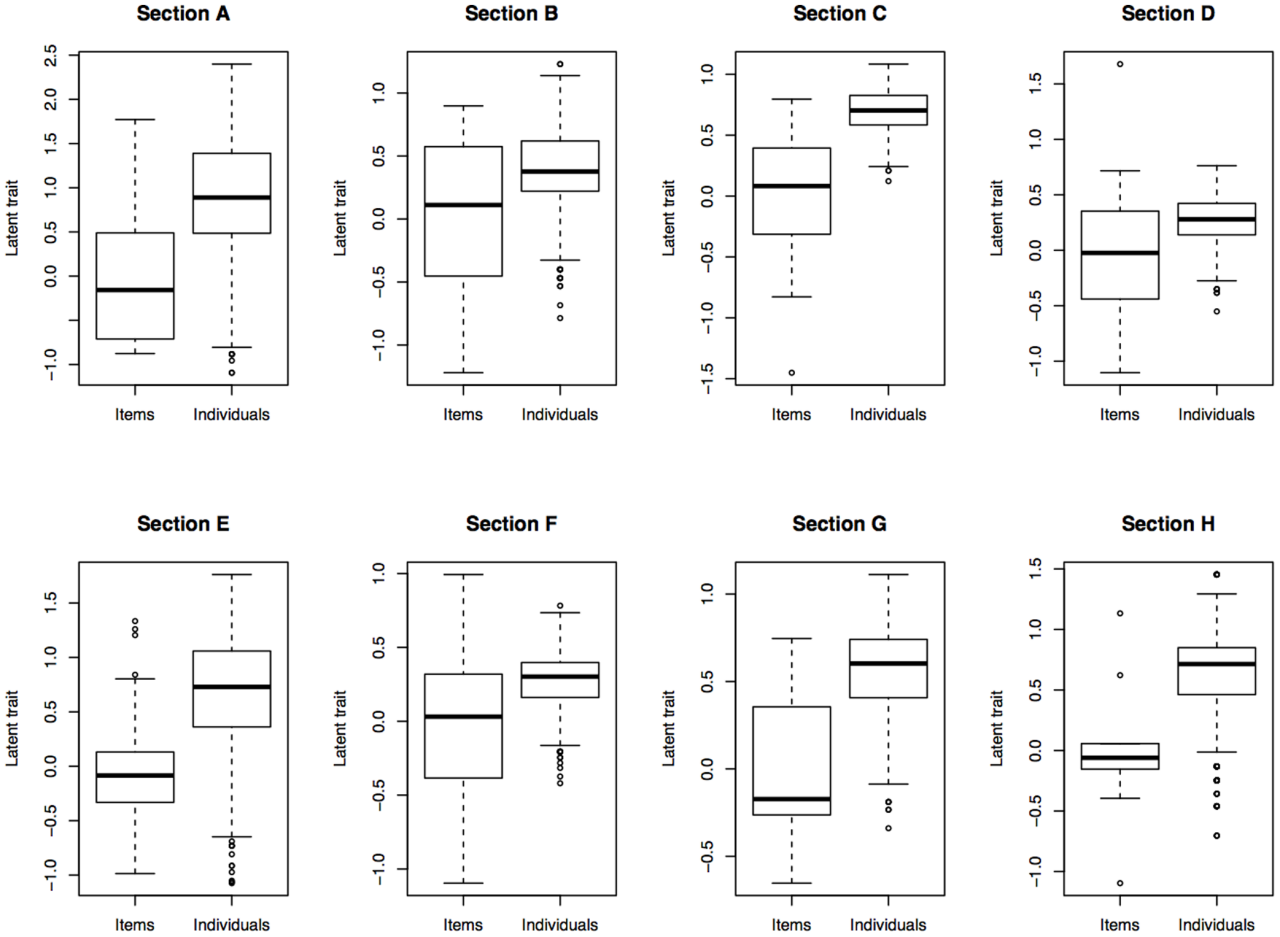
Comparison of how items and individuals are positioned on the same latent trait in the eight sections of the MSCEIT.

Figure 4 shows the location of the estimated level of the person (left side, represented by the symbol X) and the items’ difficulty (right side) on the same latent trait for the two sections of Branch 4, Managing Emotions (results for the other sections of the MSCEIT present a similar patter and are available upon request). Both higher ability individuals and more difficult items are located on the upper side, whereas low ability and low difficulty on the lower side of the vertical line. The most evident result of this comparison is that the distribution of the persons is shifted with respect to the distribution of the items. This may be interpreted as if items are not difficult enough to challenge high ability individuals. The same pattern emerged across the different sections of the MSCEIT and is more evident in the data represented in Figure 5 as box-and-whisker plot. Here the bold line inside the boxes indicates the median, and the upper and lower limit of each box respectively the upper and lower quartile. The graph also shows the maximum and minimum score and the location of outliers, indicated by the small points at the very end of the distribution.

#### The item information function

**Figure 6:**
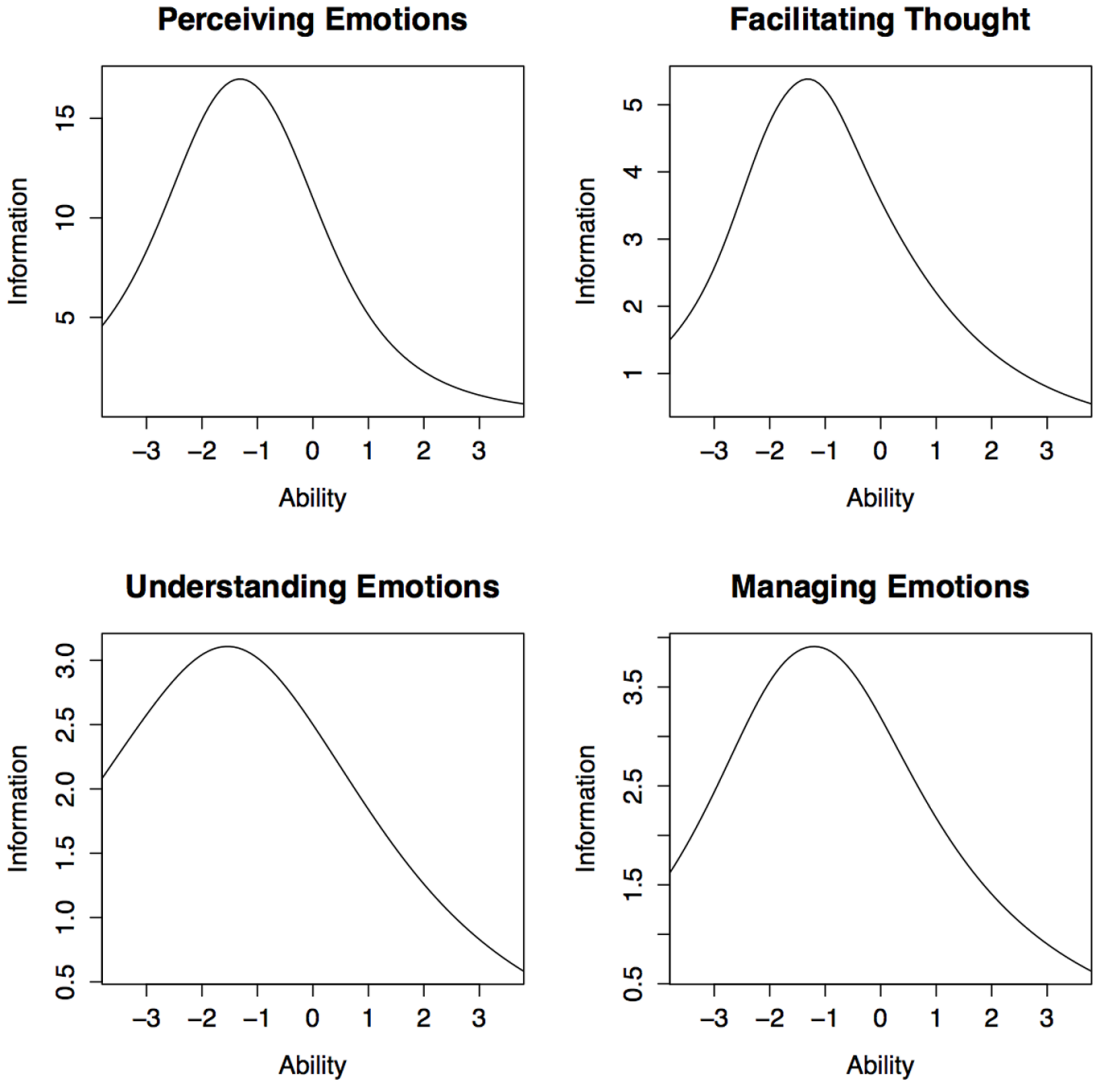
The Information Curve for each MSCEIT branches.

We calculated the amount of information provided by each item with the Item Information Function (IIF). The sum of the item response functions of a scale provides the test information function: Ij (θj) = Σi Iij (θj, bi). As previously discussed, to calculate this function we employed a more complex model in which we let the item discrimination vary from item to item. Results are shown in table 2. The percentage indicated in the table is calculated within each subsection, therefore items of subsections with more items (ex. Section E) are on average less informative than items of a subsection with fewer items (ex. Section H). Ideally each subsection should be balanced in terms of how much each item contributes to the total information of that subsection. And yet, in Section H there are two items (H8 and H9) that provide less than 1% of information. Overall items appear very heterogeneous: 12.7% of the items provide less than 1% of information, whereas 14.2% provides more than 10% of information. The information provided by each item can be summed up and plotted to describe each branch’s information function (Figure 6). From the graphs it is clear that the MSCEIT provides the most information for levels of the latent trait that are lower than 1 SD below the mean.

**Table 2:** Amount of information provided by each item as calculated with the Item Information Function.

B1-Perceiving Emotions						B2-Using Emotions						B3-Understanding Emotions						B4-Managing Emotions					
item	info	info%	item	info	info%	item	info	info%	item	info	info%	item	info	info%	item	info	info%	item	info	info%	item	info	info%
A1	0.904	2.35	E1	0.002	0	B1	2.108	14.77	F1	0.015	0.09	C1	0.609	4.75	G1	1.502	13.26	D1	2.29	16.06	H1	2.091	18.68
A2	2.198	5.7	E2	1.187	2.27	B2	1.939	13.59	F2	1.239	7.21	C2	0.702	5.47	G2	0.742	6.55	D2	1.761	12.35	H2	1.128	10.08
A3	2.497	6.48	E3	1.607	3.08	B3	0.333	2.33	F3	3.055	17.78	C3	0.192	1.5	G3	0.209	1.84	D3	1.023	7.18	H3	1.523	13.61
A4	2.101	5.45	E4	2.404	4.61	B4	0.311	2.18	F4	0.019	0.11	C4	0.578	4.51	G4	1.051	9.27	D4	0.29	2.04	H4	0.924	8.25
A5	2.151	5.58	E5	2.227	4.27	B5	0.274	1.92	F5	1.018	5.93	C5	0.201	1.57	G5	0.394	3.47	D5	0.386	2.71	H5	2.238	20
A6	0.981	2.54	E6	0.715	1.37	B6	1.408	9.87	F6	1.728	10.06	C6	0.317	2.47	G6	1.097	9.68	D6	0.052	0.36	H6	2.252	20.12
A7	0.63	1.63	E7	2.169	4.16	B7	1.191	8.35	F7	0.251	1.46	C7	0.029	0.23	G7	0.876	7.74	D7	0.261	1.83	H7	0.975	8.71
A8	2.002	5.19	E8	1.449	2.78	B8	1.518	10.64	F8	0.669	3.9	C8	0.766	5.97	G8	0.874	7.71	D8	0.968	6.79	H8	0.002	0.01
A9	2.892	7.5	E9	2.575	4.94	B9	0.008	0.06	F9	1.834	10.67	C9	0.614	4.79	G9	0.591	5.22	D9	0.918	6.44	H9	0.061	0.54
A10	2.675	6.94	E10	1.468	2.81	B10	0.062	0.43	F10	0.003	0.01	C10	0.408	3.18	G10	0.623	5.5	D10	0.004	0.03			
A11	1.127	2.92	E11	0.698	1.34	B11	1.466	10.28	F11	0	0	C11	0.038	0.29	G11	1.953	17.24	D11	0.993	6.96			
A12	0.336	0.87	E12	2.564	4.91	B12	1.354	9.49	F12	1.609	9.37	C12	0.522	4.07	G12	1.417	12.51	D12	0.02	0.14			
A13	1.621	4.21	E13	3.007	5.76	B13	0.697	4.89	F13	0.386	2.25	C13	0.735	5.73				D13	0.121	0.85			
A14	2.631	6.82	E14	2.117	4.06	B14	0.505	3.54	F14	3.911	22.77	C14	1.267	9.88				D14	1.265	8.87			
A15	2.795	7.25	E15	2.782	5.33	B15	1.096	7.68	F15	1.44	8.38	C15	1.518	11.84				D15	0.097	0.68			
A16	0.161	0.42	E16	2.066	3.96							C16	0.522	4.07				D16	0.916	6.42			
A17	1.782	4.62	E17	2.248	4.31							C17	0.693	5.4				D17	1.608	11.28			
A18	2.86	7.42	E18	1.068	2.05							C18	1.269	9.89				D18	0.508	3.56			
A19	3.157	8.19	E19	1.279	2.45							C19	0.658	5.13				D19	0.181	1.27			
A20	3.05	7.91	E20	2.3	4.41							C20	1.187	9.25				D20	0.597	4.18			
			E21	0.804	1.54																		
			E22	2.176	4.17																		
			E23	2.273	4.36																		
			E24	1.769	3.39																		
			E25	1.544	2.96																		
			E26	0.991	1.9																		
			E27	0.882	1.69																		
			E28	2.156	4.13																		
			E29	1.576	3.02																		
			E30	2.081	3.99																		

## Discussion

The purpose of the current analysis was to understand the extent to which the MSCEIT can be trusted as a measure of individual differences in EI. Results of the IRT analysis revealed that the test items are rather heterogeneous in the amount of information provided, and that the four branches seem to be better suited to discriminate individuals at the low end of the EI trait. More specifically, whereas individuals at the low levels of the trait of EI provided different answers depending on the level of the trait, individuals at the mean and high level of the trait provided the same answers to items regardless of whether they were higher or lower on EI.

### How to Use The MSCEIT In EI Research

The fact that this test does not seem to have strong measurement precision for distinguishing average from high EI individuals poses some limitations regarding how it should be employed for practice and research. Regarding its use for research, scholars employing this test should take into consideration the fact that the MSCEIT may not provide reliable results when employed with individuals that are supposed to cover the whole range of EI scores. In fact, in such cases the test would fail to detect differences among individuals that score average and above average, say on perceiving emotions, using emotions, understanding emotions, and managing emotions, providing similar scores for individuals that in fact are not on the same level of EI.

With respect to the use of the MSCEIT in applied settings, our analysis shows that this test would be appropriate for testing clinical subsamples that are expected to be below average on EI, but not for testing the normal population. MSCEIT users that employ the test for recruitment and personnel assessment should consider that this test may be effective to detect individuals with low EI, but it may not accurately discriminate average from above average individuals.

### How to Improve the Current Version of the MSCEIT

Our analysis revealed that certain aspects of the MSCEIT could definitely be improved in a revised version. The item information function (IIF) showed that about 13% of the items included in the test provide less than 1% of information. Of note, the MSCEIT manual on p. 63 states that 19 items present in the test are excluded from the test scoring. We asked MHS the list of these items: 8 correspond to items that provide less than 1% of information. Eleven items could still be removed because of the low information they convey. Given the length of the test, these items could be simply removed without impacting on the psychometric properties of the test.

Our analysis shows that several items function as dichotomous rather than as items with different nuances of response. In addition, because most individuals identified the most correct answer, the different degrees of correctness were seldom endorsed, showing that the test items are overall rather easy. Consequently, as a second recommendation we suggest to improve the current version of the test by either scoring responses as correct or wrong, or by introducing response options that would guarantee more endorsement by test takers so as to capture nuances among individuals that possess different levels of the latent trait.

A final recommendation regarding how to improve the test does not derive directly from the results of the IRT analysis, but stems from a more general consideration on the scoring system employed in the MSCEIT. Mayer, Salovey, and Caruso have always recommended the consensus-based scoring as the best option (e.g., [11]) claiming that this scoring system is the most reliable. To solve the issue raised by Roberts et al. [31] regarding the lack of convergence between expert and consensus-based scoring of the previous version of the MSCEIT, the MEIS, it seems as if the authors reacted by modifying the test in a way that caused experts and consensus ratings to converge from the original .26 of the MEIS to.98 of the current version of the MSCEIT. Perhaps their attempt to make the two systems converge was done at the expense of the quality of the items.

Mayer et al. ([13] p. 237) explained the high levels of convergence by saying that “Experts look for the correct answer by paying attention to the consensual information of the group.” However, as Maul also noticed [20], what the majority of people say about emotions may simply reflect lay theories, which, although shared by most, can still be incorrect. The ability to spot a fake smile is a good example of this effect. Maul [20] shows that this task is challenging for all but a restricted group of emotion experts. In this case a “correct” answer should be modeled on the few that can spot fake emotions, not on the modal answer in the total sample. In fact, the emotional intelligent ‘prototype’ person should be among the very few that can spot fake emotions, rather than among the vast majority of people that get them wrong. Thus, from a conceptual point of view, it would make more sense to score individuals with respect to a group that by itself could be equated with high EI individual (namely emotion experts), as long as items reflect differences between normal individuals and those that are higher than the norm. We suggest that these problems in the MSCEIT may be ameliorated by choosing items that show a certain degree of divergence (perhaps something in the middle between .26 and .98) rather than selecting those for which experts and general people provided almost the same answers.

Before concluding we would like to acknowledge certain limitations of our study. An assumption of measurement models is that correlations among items should be due only to the common latent trait. In a recent study [24] it was suggested to model variance in item response according to the stimulus material, so as to account for shared variance that depends on the structure of the test rather than on the latent trait. The idea is that if one person judges a picture as expressing a great extent of joy, then *as a consequence* this person will judge the picture as expressing very little sadness. Thus, scores on the joy and sadness items would depend at least in part on the interpretation of the picture and not exclusively on the level of Emotion Perception of the person. Maul’s recommendation certainly provides valuable inputs for further analyses of the MSCEIT. At the same time, we would like to raise the possibility that people may independently perceive such emotions in the same stimulus, very much on the line of research supporting the idea that negative and positive emotions may coexist (e.g. [32]). Moreover, the issue of item dependence would affect especially the Perceiving Emotions branch of the MSCEIT and not necessarily the whole test.

It is important to notice that the results we found, in particular those on the mismatch between difficulty of items and position of individuals with respect to the same latent trait, as well as the demonstration that the MSCEIT provides the most information for levels of the latent trait corresponding to minus 1 standard deviation, were consistently observed across the different sections of the test. Therefore our analyses can be considered comprehensive and overall informative for the overall MSCEIT.

## Conclusions

Recently Maul claimed that “The central idea of measurement is to have a procedure sensitive to differences in the thing being measured, such that (…) different responses to different items are reflective of different levels of emotional intelligence” ([20] p. 8). Our analysis has shown that the MSCEIT’s items may capture differences in individuals only when such individuals are positioned at the low end of the EI trait distribution. For the other individuals (medium and high in EI) variation in the scores does not reflect true variation in EI. Given that most of the evidence collected up to date on the topic of ability EI is based on the employment of this test, and that the debate on the legitimacy of the EI construct has often taken this test as its flagship, our results warrant close consideration. We believe that understanding what aspect of EI the MSCEIT measures and how it measures it is of primary importance for advancing research in this domain.

Mayer and Salovey should be commended for having introduced the theoretical bases of EI and for having brought the study of EI on the scientific ground. We believe the domain of EI could be enhanced by better discerning the good from the less good of current research so as to build future theorization on solid foundations. After all, EI is still in its early developmental stage and it is especially at this scientific age that learning from mistakes is vital. We hope to have provided a constructive approach to one of the important issues surrounding EI, namely the extent to which scholars may rely on the MSCEIT to measure EI, and that future research will benefit from our contribution to build on the next generation of measures of EI.
